# Primary bulbar urethral adenocarcinoma arising from intestinal-type villous adenoma: a rare malignant entity

**DOI:** 10.1016/j.eucr.2026.103519

**Published:** 2026-06-23

**Authors:** Rachel Cockburn, Jennifer Xu, Hans Goossen

**Affiliations:** a Department of Urology, Queen Elizabeth II Jubilee Hospital, Metro South Health, Brisbane, Australia; b Faculty of Medicine, University of Queensland, Australia

**Keywords:** Urethral adenocarcinoma, Intestinal-type, Urethral stricture disease, Subtotal urethrectomy, Perineal urethrostomy

## Abstract

Primary urethral adenocarcinoma is rare, with limited guidance for management. A 65-year-old male presented with haematuria and urinary retention, found to have a bulbar urethral tumour at the site of previous stricture. Histology demonstrated moderately differentiated adenocarcinoma arising from an intestinal-type villous adenoma with high-grade dysplasia. Staging excluded metastatic or alternative primary disease. He underwent subtotal urethrectomy with perineal urethrostomy, achieving clear margins. Mild postoperative stenosis was managed conservatively. At one year, there is no recurrence. This case describes gastrointestinal adenocarcinoma in the setting of urethral stricture disease and highlights tailored surgical management in achieving favourable oncological and functional outcomes.

## Highlights

• Primary urethral adenocarcinoma is an extremely rare malignancy with limited evidence to guide management.
• Intestinal-type adenocarcinoma may arise from a metaplasia–dysplasia–carcinoma sequence.
• Urethral **stricture disease**, prior instrumentation and **chronic inflammation** may contribute to tumour development.
• Diagnosis requires exclusion of a gastrointestinal primary and metastatic disease.
• Individualised multidisciplinary management is essential in this rare tumour type.

## 1 Introduction

Primary urethral carcinoma is a rare genitourinary malignancy, accounting for less than 1% of all urological cancers. Among these, adenocarcinoma represents a small subset, with intestinal-type variants being particularly uncommon. Due to its rarity, there is limited high-quality evidence to guide diagnosis and management, and clinical decisions are often based on small case series and expert opinion. The pathogenesis remains incompletely understood, although chronic inflammation, urethral stricture disease, and repeated instrumentation have been implicated in promoting metaplastic and dysplastic changes within the urethral epithelium. Intestinal-type urethral adenocarcinoma poses additional diagnostic challenges, as it closely resembles colorectal adenocarcinoma and requires exclusion of a metastatic or secondary primary source. We present a case of intestinal-type urethral adenocarcinoma arising from a villous adenoma in the setting of chronic urethral stricture disease, highlighting its clinical presentation, pathological features, and surgical management.

## 2 Case presentation

A 65-year-old male presented with macroscopic haematuria and acute urinary retention. His medical history included obesity, obstructive sleep apnoea, and atrial fibrillation on rivaroxaban. His urological history was significant for previous transurethral resection of the prostate (TURP) in 2009 and subsequent bladder neck incision in 2010, after which he developed a bulbar urethral stricture disease requiring an optical urethrotomy in 2011. He also had a history of recurrent nephrolithiasis requiring endoscopic management.

**Fig. 1:**
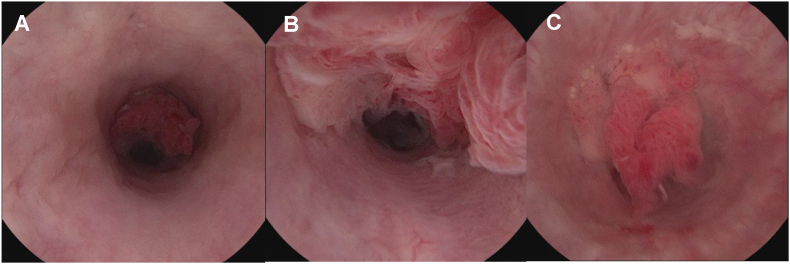
2cm papillary tumour arising from the bulbar urethra of this patient. Appearance from penile urethra (panel A). Appearance up close (panel B). Appearance macroscopically at bulbar urethra (panel C).

Endoscopic assessment demonstrated a 2cm papillary tumour in the bulbar urethra at the site of previous stricture disease (Fig. 1; panel A-C). The remainder of the urethra, prostatic fossa, and bladder mucosa were unremarkable. Transurethral resection of the urethral lesion was performed. The bladder and prostatic urethra appeared macroscopically normal. Computed tomography intravenous pyelogram (CT IVP) demonstrated no upper tract urothelial malignancy, hydronephrosis, or regional lymphadenopathy.

**Fig. 2:**
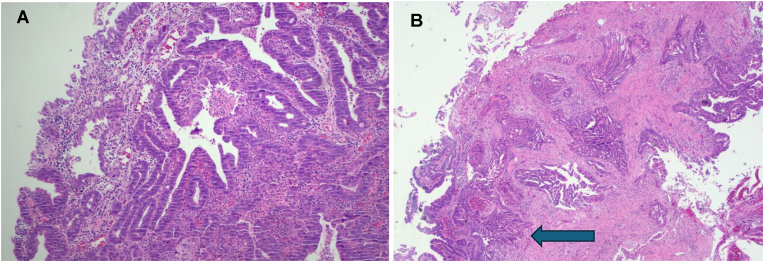
Bulbar urethral tumour histology. Complex villiform and tubular glands lined by columnar epithelium showing high grade atypia (panel A). Infiltrative malignancy with stromal reaction is seen by the arrow (panel B).

**Fig. 3:**
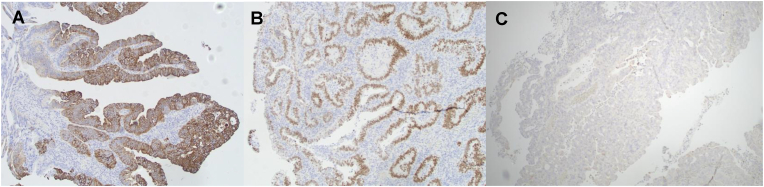
Positive immunohistochemical staining for CK20 positive (panel A) and CDX2 positive (panel B), markers used to identify gastrointestinal differentiation. Negative staining for NKX3.1 used as a marker of prostatic origin (panel C).

Histopathological examination revealed a complex villiform and tubular glands lined by columnar epithelium showing high grade atypia with infiltrative components (Fig. 2). Immunohistochemical staining was positive for the gastrointestinal markers CK20 and CDX2 (Fig. 3). Gastrointestinal immunohistochemical staining was positive for CK20 and CDX2 (Fig. 3). NKX3.1 staining was negative, helping to exclude prostatic glandular tissue as the source of the lesion (Fig. 3). The morphology and immunohistochemical findings were consistent with moderately differentiated adenocarcinoma arising from an intestinal-type villous adenoma with high-grade dysplasia, consistent with invasive urethral adenocarcinoma. Fluorodeoxyglucose positron emission tomography (FDG-PET) demonstrated no nodal or distant metastatic disease and no gastrointestinal primary, supporting a primary urethral origin.

Given the tumour's location relative to continence structures, management options were carefully considered. These included organ-preserving surgery with subtotal urethrectomy and reconstruction, radical penectomy, or non-surgical options including chemoradiation. Following multidisciplinary discussion and further discussion with patient, he underwent subtotal urethrectomy with perineal urethrostomy and rotational perineal flap urethral reconstruction.

Final histology demonstrated residual adenocarcinoma with clear surgical margins. Postoperatively, the patient developed mild perineal urethrostomy stenosis, managed with intermittent self-dilatation. He remains under computed tomography surveillance with no evidence of disease recurrence at 10 months post-operatively.

## 3 Discussion

Primary urethral carcinoma is extremely rare, comprising less than 1% of all urological cancers. Adenocarcinoma accounts for fewer than 5% of urethral tumours.^1^ Histologically, urethral adenocarcinoma is subdivided into clear cell and columnar or mucinous (intestinal) subtypes. The intestinal variant closely resembles colorectal adenocarcinoma and may present diagnostic challenges in distinguishing primary urethral disease from secondary involvement.^2^

Several theories regarding tumour origin have been proposed. One theory of tumour origin involves malignant transformation from intestinal metaplasia or urethritis glandularis in the setting of chronic mucosal irritation,^2^ while another implicates periurethral glandular tissue as the cell of origin.^3^ In this case, the tumour developed at the site of longstanding urethral stricture disease following multiple endoscopic procedures. Chronic inflammation and repeated instrumentation may have created a pro-inflammatory microenvironment conducive to metaplastic change and subsequent malignant transformation. Several case reports have described the development of squamous cell carcinoma in patients with urethral stricture disease, with chronic inflammation thought to drive malignant pathogenesis.^4^^,^^5^ However, the gastrointestinal differentiation observed in our case remains difficult to explain. The presence of intestinal-type villous adenoma with high-grade dysplasia in this case supports a metaplasia–dysplasia–carcinoma sequence analogous to colorectal tumorigenesis,^6^ although the factors driving this within the urethra remain rare and uncertain.

Presentation is often non-specific, contributing to diagnostic delay, with many patients presenting with locally advanced disease at diagnosis.^1^ Consequently, prognosis remains guarded,^7^ with reported five-year survival rates of approximately 30%.^2^

Management is largely extrapolated from small case series.^1^^,^^2^^,^^8^ For small, distal, non-invasive lesions, organ-preserving approaches such as local excision or segmental urethrectomy may be appropriate.^2^ However, more proximal or invasive disease often necessitates aggressive surgical resection, including subtotal or total urethrectomy, with or without urinary diversion.^1^^,^^8^ The role of chemotherapy and radiotherapy remains uncertain,^2^ with no prospective trials guiding treatment decisions.

## 4 Conclusion

Primary intestinal-type urethral adenocarcinoma remains an exceptionally rare malignancy with limited evidence to guide management. This case demonstrates that, in carefully selected patients with localised disease, surgical management can achieve effective oncological control while preserving continence and penile function. The findings also support a potential role for chronic urethral inflammation and prior instrumentation in tumour pathogenesis. Early recognition, thorough exclusion of an alternative primary, and careful pathological assessment are critical to diagnosis. Given the absence of prospective data, multidisciplinary input and individualised surgical planning remain essential in optimising both oncological and functional outcomes.

## CRediT authorship contribution statement

**Rachel Cockburn:** Writing – original draft, Methodology, Investigation, Data curation. **Jennifer Xu:** Writing – review & editing, Conceptualization. **Hans Goossen:** Writing – review & editing, Conceptualization.

## Patient consent

Written informed consent was obtained from the patient for publication of this case report and accompanying images.

## Funding

This research did not receive any specific grant from funding agencies in the public, commercial, or not-for-profit sectors.

## Declaration of competing interest

The authors declare that they have no known competing financial interests or personal relationships that could have appeared to influence the work reported in this paper.
